# Hemophagocytic Lymphohistiocytosis in an AIDS Patient with Kaposi Sarcoma: A Treatment Dilemma

**DOI:** 10.1155/2019/7634760

**Published:** 2019-10-07

**Authors:** Oluwadunni E. Emiloju, Sorab Gupta, Vivian Arguello-Guerra, Claudia Dourado

**Affiliations:** ^1^Department of Medicine, Einstein Medical Center, Philadelphia, PA 19141, USA; ^2^Department of Hematology and Medical Oncology, Einstein Medical Center, Philadelphia, PA 19141, USA; ^3^Department of Pathology and Laboratory Medicine, Einstein Medical Center, Philadelphia, PA 19141, USA

## Abstract

Hemophagocytic lymphohistiocytosis (HLH) is a result of an abnormal activation of immune cells (T lymphocytes, natural killer cells, and macrophages) resulting in cytokine overproduction and immune destruction of cells, eventually resulting in multiorgan failure. Genetic causes are responsible for primary hemophagocytosis, but malignancies, infections, and autoimmunity underlie most of the secondary cases. We present an unusual case of a patient with AIDS and disseminated Kaposi sarcoma who was commenced on highly active antiretroviral therapy (HAART) but developed HLH secondary to immune reconstitution inflammatory syndrome (IRIS). We report this case to highlight the difficulty in managing this patient given the complex interplay of immunosuppression due to AIDS, immune reconstitution following initiation of HAART, and immune overdrive manifesting as HLH.

## 1. Introduction

Hemophagocytic lymphohistiocytosis (HLH) is a result of an abnormal activation of immune cells (T lymphocytes, natural killer cells, and macrophages) resulting in cytokine overproduction and immune destruction of cells, eventually resulting in multiorgan failure. Primary HLH is due to genetic defects and usually presents in childhood and very rarely in adults. However, secondary HLH can be triggered by a number of conditions including infections (viral, bacterial, fungal, and parasitic infections), malignancies (particularly lymphomas), immunodeficiencies, and autoimmune conditions.

Kaposi sarcoma is an AIDS-defining illness, and the cornerstone of AIDS-related Kaposi sarcoma treatment is highly active antiretroviral therapy (HAART).

We present a case of a patient with disseminated Kaposi sarcoma who was commenced on HAART but developed fatal hemophagocytosis secondary to immune reconstitution inflammatory syndrome (IRIS). We report this case to highlight the difficulty in managing this patient given the complex interplay of immunosuppression due to AIDS, immune reconstitution following initiation of HAART, and immune overdrive manifesting as HLH.

## 2. Case Description

A 59-year-old female with a past medical history of hypothyroidism presented with a rash involving her scalp, neck, torso, and vagina. She denied taking any new medication and had been on levothyroxine replacement for about 12 years: she had no known allergies. There was no significant family history; she was an ex-cigarette smoker with a 20-pack-year smoking history. Physical examination was significant for diffuse purplish plaques over the torso. Human immunodeficiency virus (HIV) viral load was 196,000 copies/ml with CD4 count 76/*μ*L. Biopsy of the rash revealed Kaposi sarcoma, and she was commenced on HAART (emtricitabine, tenofovir alafenamide, and dolutegravir), trimethoprim-sulfamethoxazole, and fluconazole prophylaxis for opportunistic infections.

**Figure 1:**
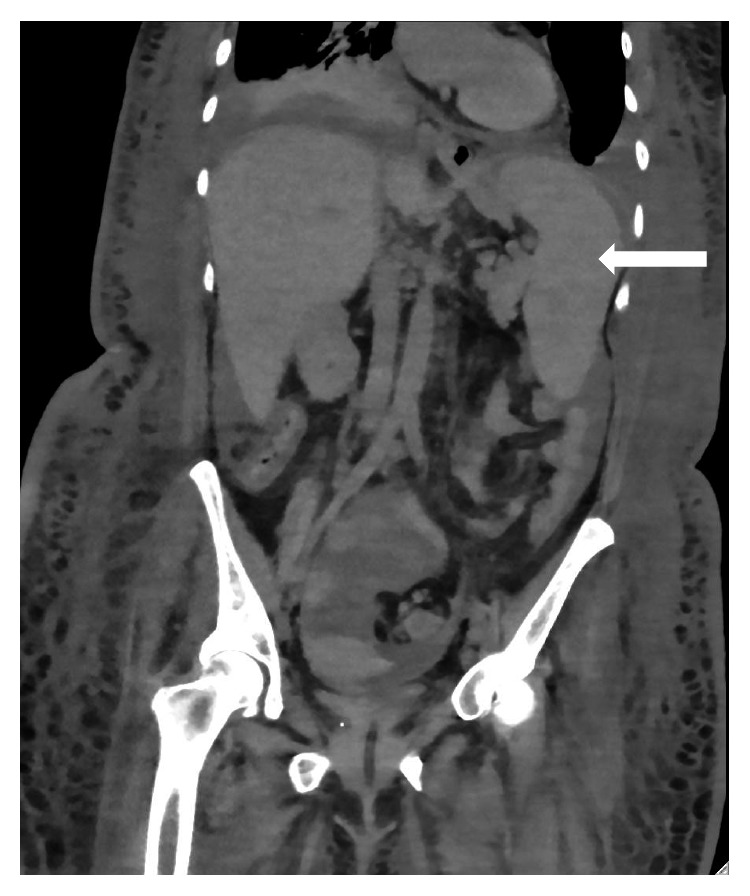
CT scan of the abdomen and pelvis. Abdominal CT scan showing splenomegaly. The spleen was enlarged and was 14 cm in length (white arrow pointing to the enlarged spleen).

She re-presented two weeks later with fever. Physical examination revealed a maximum temperature of 38.8°C and tachycardia (pulse rate 108/min), and previously noted cutaneous Kaposi lesions were still present. She was worked up with appropriate cultures and serological testing, but no opportunistic infection was found. Further workup of her Kaposi sarcoma including upper gastrointestinal endoscopy and CT scan of her thorax, abdomen, and pelvis revealed no visceral involvement, but splenomegaly was present (Figure 1). Her fever resolved without antibiotics, but fatigue persisted, and this was attributed to HIV-associated cytopenias (platelet count 86,000/*μ*L and hemoglobin 6.6 g/dl) for which she received red cell transfusion with improvement in her hemoglobin level (hemoglobin 9.0 g/dl after receiving 2 units of red blood cells). Her HAART regimen remained uninterrupted, and she was discharged. Follow-up labs 1 week after discharge showed worsening cytopenia (platelet count 50,000/*μ*L and hemoglobin 8.4 g/dl), and she was referred to the Hematology office for evaluation.

**Figure 2:**
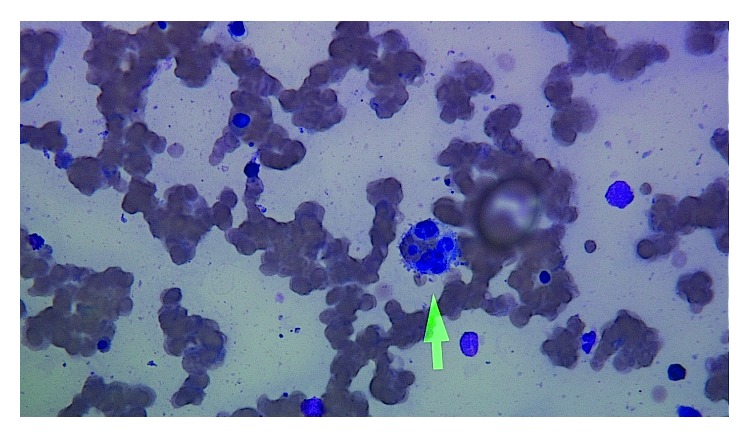
Bone marrow aspirate smear. The Wright Giemsa stain of the patient's bone marrow aspirate with an arrow highlighting a macrophage phagocytizing red blood cells, lymphocytes, and neutrophils (arrow pointing to the macrophage).

She again presented 1 month from AIDS diagnosis with severe fatigue and diarrhea to another hospital. She was found to be hypotensive (systolic blood pressure 80 mmHg), necessitating intravenous fluids and a brief course of vasopressors before she was transferred to our institution. Initial labs showed pancytopenia (platelet count 6,000/*μ*L, hemoglobin 4.3 g/dl, and leucocytes 1,900/*μ*L). Her HIV viral load had improved from 196,000 copies/ml to 670 copies/ml, CMV antibody and parvovirus IgM were negative, and no bacterial or fungal infections were detected on cultures. She was supported with transfusions of red blood cells and platelets, but her response to transfusion was suboptimal, necessitating multiple transfusions. She also received intravenous immunoglobulin (1 g/kg), but her cytopenias persisted. She continued to receive intravenous immune globulin (IVIG) and high-dose steroids, but her pancytopenia worsened necessitating a bone marrow biopsy. Her bone marrow aspirate showed hemophagocytosis (Figure 2). Further testing revealed splenomegaly on CT scan of her abdomen, ferritin of 2,568 ng/mL, triglycerides of 151 mg/dL, and fibrinogen of 279 mg/dL. Consideration was given to discontinuing her HAART because she was undergoing immune reconstitution leading to hemophagocytosis, but the need for treating her widespread Kaposi prevailed and HAART was continued. Cytopenia progressively worsened, and she developed liver, kidney, bone marrow, respiratory, and heart failure which led to her death.

## 3. Discussion

Hemophagocytic lymphohistiocytosis (HLH) is a fulminant disorder resulting in unbridled activation of immune cells, resulting in death if untreated [1]. Primary HLH is due to genetic defects and usually presents in childhood and very rarely in adults. However, secondary HLH can be triggered by a number of conditions including infections (viral, bacterial, fungal, and parasitic infections), malignancies (particularly lymphomas), immunodeficiencies, and autoimmune conditions [2]. Of the recognized secondary causes of HLH, viral infections such as EBV, herpesviruses, including human herpesvirus 8 (HHV8), HIV, influenza, parvovirus, and hepatitis viruses are recognized causes [3]. A retrospective study of hemophagocytic syndrome in patients living with HIV revealed that the commonly found triggers in HIV-associated HLH were mycobacterium, CMV, cryptococcus infections, and malignancy [4].

Although she had a high HIV viremia and HHV8 (Kaposi-associated herpesvirus) infection at diagnosis, hemophagocytosis was not manifest until 6 weeks after HAART was initiated: the temporal association between HAART and hemophagocytosis makes immune reconstitution inflammatory syndrome (IRIS) the most probable trigger of her hemophagocytosis.

IRIS is the restoration of the immune response to pathogen-specific antigens, resulting in unmasking of previously subclinical opportunistic infections and nonpathogen-specific response manifesting as autoimmune conditions such as Graves' disease [5]. It is not uncommon for IRIS to develop after initiation of HAART, but its exact incidence is not known [6]. In a retrospective study, 25% of HIV patients manifested opportunistic infections after commencement of HAART [7].

Patients with IRIS manifest clinical worsening despite improvement in their immune function as evidenced by improving HIV viremia and/or CD4 count. Our patient responded to HAART as evidenced by a reduction in her viremia from 196,000 RNA/mL to 670 RNA/mL, signifying a recovery of her immune system. In our patient, she already had clinically apparent extensive cutaneous Kaposi sarcoma before the initiation of HAART, and there was no clinical worsening of her skin lesions after the commencing HAART. Her clinical deterioration was secondary to hemophagocytosis.

In the medical literature, HLH is not described as part of the spectrum of immune reconstitution in the setting of HIV/AIDS. There have however been a few case reports suggesting that hemophagocytosis secondary to IRIS can occur. Two cases of hemophagocytosis secondary to IRIS in AIDS patients with lymphoma as well as an additional 2 cases of IRIS-associated hemophagocytosis in the absence of lymphoma have been reported [8–11].

Treatment modalities in HLH include immunosuppressants such as high-dose steroids, intravenous immunoglobulin, chemotherapeutic agents such as etoposide, and allogeneic hemopoietic stem cell transplant [1]. There are no established guidelines for the treatment of IRIS-associated HLH, but previous case reports have employed continued HAART, steroids, and intravenous immunoglobulin (IVIG) [8–11]. In a retrospective review of HIV-associated hemophagocytosis, patients were also continued on HAART [12].

Given the heterogeneity of the clinical manifestations of IRIS, treatment strategies also vary and are usually aimed at the underlying infection or autoimmune condition. If patients have multiple risk factors for the development of IRIS following initiation of HAART, clinicians sometimes commence treatment for the underlying opportunistic infection first with a lag period before initiating HAART [6]. In most cases of IRIS, HAART is continued while disease-specific treatment along with anti-inflammatory therapy (nonsteroidal anti-inflammatory drugs and steroids) is used [13, 14]. In severe, life-threatening IRIS, however, some clinicians are of the opinion that there may be a need for temporary discontinuation of HAART [6]. In a patient like ours, with coexisting IRIS and HLH, there may be a role for temporary discontinuation of HAART until the HLH resolves. The other important question to answer would be to determine if there is a role for the initiation of etoposide in the setting of IRIS-associated hemophagocytosis. There are however no studies that have addressed these specific questions.

In a consensus guideline issued by members of the adult HLH working group of the Histiocyte Society, etoposide is recommended for treating adult HLH, but the duration and intensity of treatment should be tailored to the severity of HLH on a case-by-case basis. In refractory HLH, combination chemotherapy and allogenic stem cell transplantation is recommended [15].

Furthermore, due to the rarity of IRIS-associated HLH, affected patients may not be diagnosed early enough. With the benefit of hindsight, our patient had bicytopenia, fever, and splenomegaly at her prior presentation, and an earlier bone marrow biopsy might have been warranted. Although there are certain criteria that must be met to diagnose HLH, patients may not develop all these features at once, and this may lead to a delay in diagnosis and treatment. One of such criteria is elevated soluble interleukin-2 receptor (sIL-2r), also referred to as soluble CD 25 (sCD 25). Although sIL-2r of at least 2400 U/mL has a high sensitivity for HLH, it was only 63% specific in the adult cohort studied by Hayden et al. [16]. Values above 10,000 U/mL had 93% specificity for ruling in HLH [16].

The commonly used criteria were developed by the HLH-2004 study group who only studied the pediatric population [17]. Other scoring systems have also been developed. A more applicable scoring system for the diagnosis of secondary HLH in adults is the HScore, given that it was developed by studying a cohort of adult patients. HScore is calculated by adding scores for several criteria including underlying immunosuppression, fever, hepatosplenomegaly, cytopenias, hyperferritinemia, hypertriglyceridemia, hypofibrinogenemia, aspartate aminotransferase level, and hemophagocytosis on bone marrow aspirate [18]. The best cutoff HScore was 169 which had 93% sensitivity and 86% specificity for diagnosing HLH in 90% of the patients in the cohort [18]. Our patient had an HScore of 241 which corresponds to 99% probability of having HLH [18]. In a retrospective study of hemophagocytic syndrome in patients living with HIV, bone marrow aspirate had 93% specificity for diagnosis, while ferritin levels greater than 5000 ng/mL correlated with increased likelihood of death [4].

There remains the need to conduct a prospective study to evaluate the efficacy of the various treatment modalities for HLH in adults.

## 4. Conclusion

Clinicians need to maintain a high index of suspicion for HLH in the appropriate clinical setting. This is because an early diagnosis will aid the prompt initiation of treatment, and this can improve patient outcomes. Regarding our patient presented here, the most challenging aspect of her care was how to manage her as there are currently no treatment guidelines for IRIS-associated HLH in the setting of AIDS.

Future efforts need to be directed primarily towards conducting prospective studies to evaluate the efficacy of the various treatment modalities for HLH in adults. The specific subset of adults with IRIS, AIDS, and HLH can then be studied secondarily. We believe that by reporting this case, clinicians will be more aware of the possibility of HLH developing after initiating HAART in AIDS patients.
